# p16^INK4a^ Translation Suppressed by miR-24

**DOI:** 10.1371/journal.pone.0001864

**Published:** 2008-03-26

**Authors:** Ashish Lal, Hyeon Ho Kim, Kotb Abdelmohsen, Yuki Kuwano, Rudolf Pullmann, Subramanya Srikantan, Ramesh Subrahmanyam, Jennifer L. Martindale, Xiaoling Yang, Fariyal Ahmed, Francisco Navarro, Derek Dykxhoorn, Judy Lieberman, Myriam Gorospe

**Affiliations:** 1 Laboratory of Cellular and Molecular Biology, National Institute on Aging-IRP, National Institutes of Health (NIH) NIH, Baltimore, Maryland, United States of America; 2 CBR Institute for Biomedical Research, Department of Pediatrics, Harvard Medical School, Boston, Massachusetts, United States of America; Victor Chang Cardiac Research Institute, Australia

## Abstract

**Background:**

Expression of the tumor suppressor p16^INK4a^ increases during aging and replicative senescence.

**Methodology/Principal Findings:**

Here, we report that the microRNA miR-24 suppresses p16 expression in human diploid fibroblasts and cervical carcinoma cells. Increased p16 expression with replicative senescence was associated with decreased levels of miR-24, a microRNA that was predicted to associate with the p16 mRNA coding and 3′-untranslated regions. Ectopic miR-24 overexpression reduced p16 protein but not p16 mRNA levels. Conversely, introduction of antisense (AS)-miR-24 blocked miR-24 expression and markedly enhanced p16 protein levels, p16 translation, and the production of EGFP-p16 reporter bearing the miR-24 target recognition sites.

**Conclusions/Significance:**

Together, our results suggest that miR-24 represses the initiation and elongation phases of p16 translation.

## Introduction

In mammalian systems, the maintenance of homeostasis requires a tight control of cell proliferation. p16^INK4a^ (hereafter p16) inhibits the cyclin-dependent kinases CDK4 and CDK6, thereby keeping the retinoblastoma protein (pRB) in a hypophosphorylated state and arresting cells in the G1 phase of the division cycle [1]. Loss or inactivation of the *INK4a/ARF* locus (harboring p16 and ARF) are among the most frequent alterations seen in human cancers, underscoring the widely recognized role of p16 as a tumor suppressor [2], [3]. In addition, p16 critically influences the process of *replicative senescence*, whereby cells reach a state in which they remain metabolically active but appear incapable of further division. Cellular senescence is triggered by stimuli such as aberrant proliferative signals or damage to DNA or other macromolecules [4], [5]. In agreement with earlier observations that p16 levels increased with aging [6]–[8], and that p16 overexpression triggers a senescent phenotype [9] recent discoveries support the notion that cellular senescence plays a direct role in mammalian aging. In mouse models, p16 deficiency partially prevented the age-induced decline in cell proliferation and tissue function [10].

Given the prominent role of p16 in cancer and senescence/aging, there is much interest in understanding the molecular regulators of p16 expression as triggered by both environmental signals and developmental cues. Transcriptionally, CDC6 was reported to silence the *INK4a/ARF* locus by heterochromatinization linked to DNA replication [11]. In addition, p16 expression was shown to be induced by transcription factors such as Ets1, Ets2, and JunB [12], [13] and repressed by transcription factors such as Id1 and Id3 [13], [14] and by Bmi-1 [15]. Posttranscriptionally, the splicing of p16 mRNA was proposed to be influenced by ASF/SF2 and the p16 mRNA stability reduced by RNA-binding proteins (RBPs) hnRNP A1, hnRNP A2, and AUF1 [16], [17].

RBPs constitute a major class of major *trans* acting factors that associate with RNAs and regulate gene expression after transcription by influencing processes such as pre-mRNA splicing and maturation, and mRNA transport, storage, stability, and translation [18]. The other major group of *trans* acting, posttranscriptional regulatory factors are microRNAs (miRNAs), a collection of short (∼21–26-nt long), single-strand, noncoding RNAs that have been described in a wide variety of organisms. The molecular details of miRNA-mediated suppression of gene expression (*silencing*) are rapidly emerging. miRNAs are assembled into RNA-induced silencing complexes (RISC), which recruit a target mRNA to processing bodies (P-bodies) that function as cytoplasmic foci of mRNA degradation and translational repression (reviewed in reference [19]). Depending upon the degree of complementarity of the mRNA-miRNA complex, miRNAs can promote the cleavage of the target mRNA, an occurrence that is favored by extensive complementarity with the mRNA, or they can suppress mRNA translation but not mRNA degradation in instances of less complete base-pairing [20]–[22]. However, the precise mechanisms by which miRNAs inhibit translation remains controversial. Some studies indicate that miRNAs block the initiation of translation, in some instances triggering the formation of large ribonucleoprotein (RNP) ‘pseudo-polysomes’, yet others suggest that miRNAs suppress post-initiation steps by repressing translational elongation, by promoting ribosome drop-off or by inhibiting termination [23]–[26]. It is likely that multiple mechanisms are operating that depend on the nature of the miRNA, the untranslated regions, and the cellular context. An integrated model of miRNA action was proposed whereby miRNA action involved both translational suppression and accelerated decay [19].

Here, we have identified miR-24-2 (hereafter miR-24) as a microRNA that suppresses p16 translation in cultured human cells. Modulation of miR-24 levels by transfection of either pre-miR-24 or antisense (AS)-miR-24 directly affected p16 expression levels by altering the engagement of p16 mRNA with the translation machinery and consequently p16 biosynthesis. Our results are consistent with a role for miR-24 in repressing both the initiation and elongation stages of p16 translation.

## Results and Discussion

### Concomitantly Elevated p16 and Reduced miR-24 Levels in Senescent WI-38 HDFs

**Figure 1:**
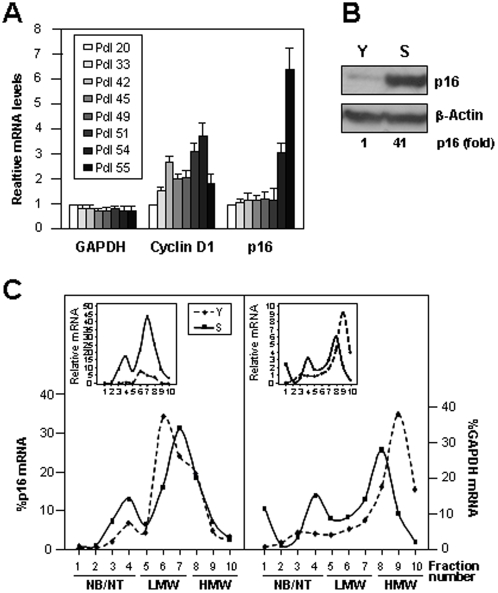
p16 levels increase with replicative senescence. (A) The levels of mRNAs encoding cyclin D1, p16, and control housekeeping GAPDH were measured by RT-qPCR, normalized to 18S rRNA levels and plotted as fold levels relative to the levels in pdl 20 HDFs. (B) Western blot analysis of p16 and loading control β-Actin levels in young (Y, pdl 20) and senescent (S, pdl 54) WI-38 HDFs. (C) The polysomal association of mRNAs encoding p16 (*left*) or GAPDH (*right*) in Y and S WI-38 cells was tested by fractionating cytoplasmic lysates through sucrose gradients and measuring mRNA abundance by RT-qPCR in each of the 10 resulting fractions (*insets*). The relative abundance of p16 and GAPDH mRNAs in each gradient fraction is shown as a percent of the total mRNA. *NB/NT* (‘not bound/not translated’) fractions with mRNA devoid of translational components; *LMW*, *HMW*, low and high molecular weight polysome fractions, respectively.

p16 protein and mRNA levels increased markedly as early-passage, proliferating (young, *Y*) WI-38 human diploid fibroblasts (HDFs) progressed towards senescence (*S*) by increasing population doublings (pdls) in culture (Fig. 1A,B). Since senescent cells showed a lesser increase in p16 mRNA abundance (>7-fold) than in p16 protein levels (∼40-fold), as previously observed [27], we hypothesized that p16 protein translation might be selectively favored with senescence. To test this possibility, cytoplasmic lysates prepared from Y or S WI-38 cells were centrifuged through sucrose density gradients in order to fractionate the translation machinery components according to their molecular weight. By this process, the lightest cytoplasmic components, which are not engaged in translation (mRNA not bound to ribosomes, and 40S and 60S ribosome subunits), appear at the top of the gradient (fractions 1–4: *NB/NT, not bound/not translated*); heavier components, comprising mRNAs bound to one or several ribosomes, appear next, in the low molecular weight (*LMW*) polysome fractions (fractions 5–7); and cosedimenting lower in the gradient (fractions 8–10) are the mRNAs considered to be most actively translated since they form part of high molecular weight (*HMW*) polysomes. The distribution of p16 mRNA (and that of the housekeeping GAPDH mRNA) was compared between Y (pdl 20) and S (pdl 54) cells. Unexpectedly, despite the low p16 protein levels in Y cells (Fig. 1A), most of the p16 mRNA was present in the LMW and HMW polysome fractions, as was seen in S cells (Fig. 1C), although the absolute p16 mRNA levels on the gradient were significantly lower in Y cells (Fig. 1C, *left*, *inset*). This observation suggested that in Y cells, while p16 mRNA associated extensively with the translational machinery, it was not actively translated, possibly through an inhibition of translational *elongation*. In addition, we noted a small but consistent shift in the p16 mRNA of Y cells towards LMW polysomes, further indicating that translational *initiation* may also be diminished. Together, these observations suggest that p16 translation could be inhibited in Y WI-38 cells, likely through a combination of decreased translation initiation and elongation.

**Figure 2:**
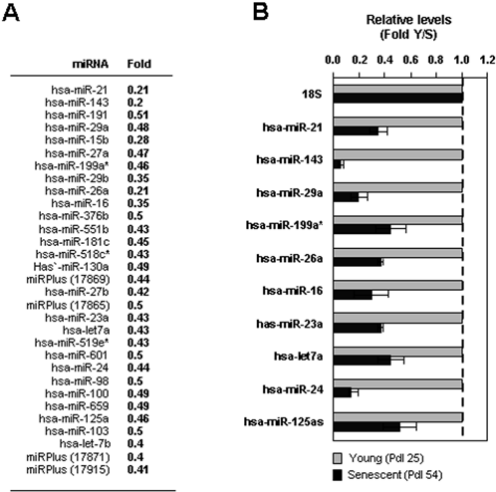
Microarray analysis of miRNAs showing reduced expression with senescence. (A) Three independent preparations of total RNA from early-passage (Young, pdl 25) and from late-passage (Senescent, pdl 54) WI-38 cells were subjected to microarray analysis (Exiqon, Materials and Methods). Thirty-one miRNAs showed reduced levels in the senescent cultures (by two-fold in at least two samples), 9 miRNAs showed increased levels (Supplemental Fig. S2). (B) The differential expression of ten downregulated miRNAs was validated using RT-qPCR analysis; the relative levels of each miRNA in Y and S WI-38 cells are shown (means+SEM from three independent experiments).

**Figure 3:**
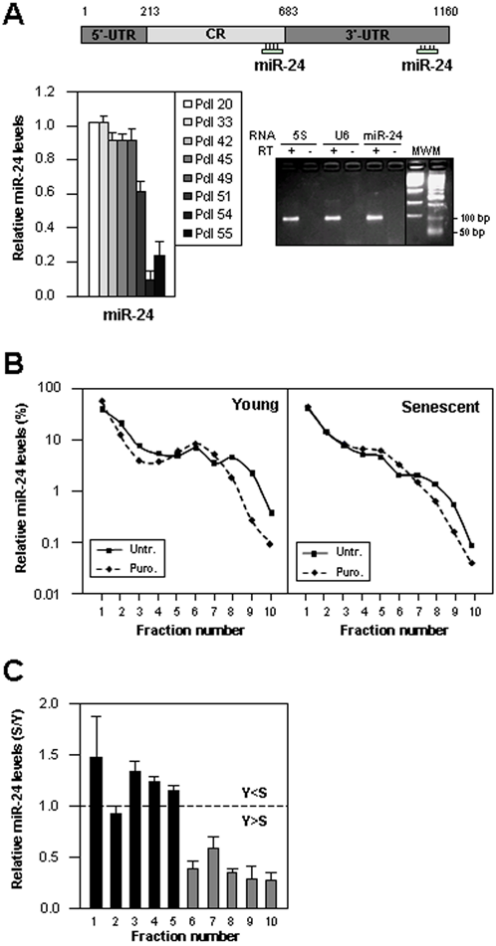
miR-24 levels decrease with replicative senescence. (A) *Top*, p16 mRNA depicting the two locations (in CR and 3′UTR) where miR-24 is predicted to bind. *Bottom left*, miR-24 levels were measured in WI-38 cells by RT-qPCR using miR-24-specific primers, and normalized to 5S rRNA levels. *Bottom right*, PCR-amplified products (∼100-bp), visualized on 1.5% agarose gels, were absent from RT- reactions; *MWM*, DNA molecular weight markers. (B) Young (pdl 20) or Senescent (pdl 54) WI-38 cells were left utnreated (Untr.) or treated with puromycin (Puro., 200 µM, 1 hr) and cytoplasmic lysates were prepared and fractionated through sucrose gradients. The presence of miR-24 was measured in each fraction, calculated as a percentage of the total miR-24, and plotted using a semilogarithmic scale. The data are representative of 3 independent experiments. (C) The relative abundance (fold) of miR-24 in each fraction was compared between Y and S cells. In fractions 1–5 (black bars), miR-24 was comparable or more abundant in S than in Y cells; in fractions 6–10 (gray bars), miR-24 was more abundant in Y than in S cells. All bars represent the means+SD from 3 independent experiments.

To test these possibilities, we examined whether RBPs implicated in translational control (like TIAR, TIA-1 or elav/Hu RBPs) bound the p16 mRNA, but no such pdl-dependent RNP interactions were observed (not shown). Therefore, we hypothesized that the translation of p16 mRNA might be influenced by its association with microRNAs. RNA was prepared from Y and S and used for miRNA microarray analysis (Exiqon, Materials and Methods); the complete array report is available from the authors. As shown (Fig. 2A), thirty-one miRNAs were found to be lower in S populations. Importantly, all of the miRNAs for which we were able to amplify PCR products (ten in total) were found to be downregulated in S cells, supporting the accuracy of the microarray analysis (Fig. 2B). The microRNA miR-24 was predicted to bind to the p16 mRNA both in the coding region (CR) and the 3′-untranslated region (UTR) (Fig. 3A), based on analysis using the Miranda and RNA22 programs ([28] Supplemental Fig. S1). Miranda also predicted miR-337 to associate with p16 mRNA, but miR-337 levels were not found to be altered during senescence (Fig. 2A). An additional nine miRNAs that were more highly expressed in S cells (Supplemental Fig. S2) will be investigated separately.

We thus set out to investigate whether miR-24 might contribute to regulating p16 expression during replicative senescence. Using miR-24-specific primer pairs and reverse transcription (RT) followed by real-time quantitative (*q*) PCR analysis, we first measured the levels of mature miR-24 (Materials and Methods) in Y and S populations (Fig. 3A, graph). On sucrose gradients, miR-24 was found to be vastly more abundant [>70% in both Y (pdl 20) and S (pdl 54) HDFs] in fractions 1 and 2, and hence dissociated from any ribosome components (Fig. 3B). However, the miR-24 that co-sedimented with polysomal fractions did appear to associate with actively translating mRNAs, since treatment with puromycin (Puro.) shifted the miR-24 distribution towards lower molecular weight fractions relative to untreated (Untr.) cells, particularly in Y populations (Fig. 3B). A direct comparison of miR-24 levels in each gradient fraction showed that in Y cells, miR-24 was relatively more abundant in the heavy fractions and less in the light fractions, while in S cells, miR-24 levels were relatively higher in the light fractions and lower in the heavy, polysome-associated fractions (Fig. 3C). This differential distribution of miR-24 further supported the view that miR-24 might contribute to an inhibition of p16 translation in Y cells that was relieved in S cells.

### miR-24 Influences p16 Expression in Young and Senescent WI-38 HDFs

**Figure 4:**
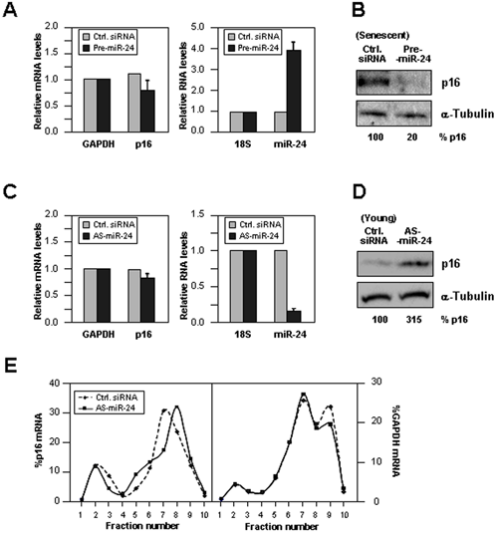
Modulation of miR-24 alters p16 levels in WI-38 HDFs. Late-passage WI-38 cells (pdl 51) were transfected with pre-miR-24 or Ctrl. siRNA (100 nM) and 48 hr later (A) RT-qPCR was used to measure the levels of p16 mRNA (normalized to GAPDH mRNA levels, *left*) and the levels of miR-24 (normalized to 18S rRNA levels, *right*), and (B) the levels of p16 and loading control α-Tubulin were monitored by Western blotting, and quantified (%) by densitometry. Early-passage (pdl 20) WI-38 cells were transfected with AS-miR-24 or Ctrl. siRNA (100 nM) and 48 hr later (C) RT-qPCR was used to measure the levels of p16 mRNA (normalized to GAPDH mRNA levels, *left*) and the levels of miR-24 (normalized to 18S rRNA levels, *right*), and (D) p16 and loading control α-Tubulin levels were assessed by Western blotting, and quantified by densitometry. (E) p16 mRNA levels in sucrose gradients were calculated as described for Fig. 1C. (A) and (C) show the means+SD from 3 independent experiments.

Next, we investigated if miR-24 directly regulated p16 protein abundance in WI-38 HDFs. The expression levels of miR-24 in WI-38 cells were modulated in both directions, elevated by using a pre-miR-24 RNA, reduced by using an antisense (AS)-miR-24 RNA. Pre-miR-24 was first introduced into late-passage cells (∼pdl 51) in order to ectopically elevate the levels of miR-24, which are naturally low in cells of advanced pdls (Fig. 3A). Despite the pre-senescent phenotype of these cells and their high p16 levels, miR-24 overexpression (Fig. 4A, *right*) markedly reduced p16 protein abundance (Fig. 4B), but not p16 mRNA levels (Fig. 4A, *left*). Conversely, Y HDFs were transfected with AS-miR-24 in order to reduce the function of miR-24 in these populations with high endogenous miR-24 levels (Fig. 3A). The intervention to reduce miR-24 (Fig. 4C, *right*) caused no significant changes in p16 mRNA levels (Fig. 4C, *left*), but it strongly increased p16 protein abundance (Fig. 4D). Transfection of AS-miR-24 caused a small but consistent shift in the peak distribution of p16 mRNA on sucrose gradients (Fig. 4E). Nonetheless, this shift occurs in the polysomal compartment (not in ligher fractons of the gradient), in agreement with the view that the translational *elongation* of p16 mRNA may be enhanced in the AS-miR-24 transfection group. Accordingly, the data could be interpreted as evidence that miR-24 exerts pressure on translating ribosomes to abort translation and ‘drop-off’, as suggested by Petersen and co-workers [24]. Together, these results support the view that the miR-24-elicited suppression of p16 translation is relieved as miR-24 levels are diminished in WI-38 HDFs progressing towards senescence.

Since AS-miR-24 elevated p16 levels in both HeLa and WI-38 cells, and p16 functions as a potent inhibitor of cell proliferation in the presence of an intact Rb pathway (such as in WI-38 cells), we hypothesized that AS-miR-24 might elicit growth arrest and possibly enhance the senescent phenotype of HDFs. These possibilities were tested in WI-38 cells following AS-miR-24 transfection. Forty-eight hr after transfection, we measured ^3^H-thymidine incorporation; against our prediction, WI-38 cells in the AS-miR-24 transfection group showed *elevated* DNA synthesis (Supplemental Fig. S3A). Moreover, following an extended transfection protocol (cells were sequentially transfected with AS-miR-24 for two weeks), the number of cells staining positive for the senescence-associated (SA) β-galactosidase activity (a marker of replicative senescence) did not increase but instead *decreased* in the AS-miR-24 treatment group (Supplemental Fig. S3B). Conversely, overexpression of miR-24 (which reduced p16 levels, as shown in Fig. 4B), did not trigger the expected increase in proliferation, but instead reduced it (Supplemental Fig. S4A); similar findings were obtained using cervical carcinoma cells (HeLa) and liver carcinoma cells (HepG2, not shown). Furthermore, extended overexpression of miR-24 after a two-week period of sequential transfections, increased SA-β-galactosidase activity (Supplemental Fig. S4B), instead of decreasing it, as anticipated. Delivery of Pre-miR-24 by using a lentiviral vector also failed to reduce the senescence phenotype (not shown).

The absence of a senescent phenotype was disappointing, but it illustrated critical aspects of the analysis and interpretation of microRNA data. A single miRNA can regulate many transcripts, possibly hundreds or thousands of transcripts. Thus, to expect a strictly linear sequence of events [single microRNA change → single altered protein → single phenotypic alteration] would be to disregard the exquisite complexity of miRNA regulatory networks. In the case of miRNA networks influencing cellular senescence, three observations can be made. First, miR-24 is predicted to bind to transcripts encoding proliferative proteins such as H-Ras (not shown), proteins acting downstream of p16, like CDK6 (Supplemental Fig. S5) and E2F2 (not shown), and also p14ARF, which shares much of the p16 mRNA sequence and is thus similarly inhibited by miR-24 (Supplemental Fig. S6). A list of additional targets of miR-24 is available from the authors. Second, p16 protein levels increase dramatically in S cells (by >40 fold, Fig. 1B), but much of this elevation is elicited by heightened p16 mRNA levels (Fig. 1A). The translational influence of modulating miR-24 levels only achieves ∼3- to 5-fold differences in p16 abundance (Fig. 4), far from the magnitude of change observed with replicative senescence. Thus, the relatively modest changes in p16 mediated by altering miR-24 levels are likely insufficient to recapitulate the influence of p16 changes occurring during senescence. Third, the process of replicative senescence is accompanied by many senescence-associated changes in the levels of numerous other miRNA, as shown in Fig. 2A. The influence of these miRNAs upon replicative senescence, as well as the influence of miR-24 upon additional targets which might impact on the senescence/proliferative phenotype of WI-38, both await further analysis. Instead, we set out to gain molecular insight into the regulation of p16 expression levels by miR-24. To this end, we employed another cell system that was amenable to interventions requiring large amounts of cells, as described below.

### Reduced p16 Expression by Ectopic Overexpression of miR-24

**Figure 5:**
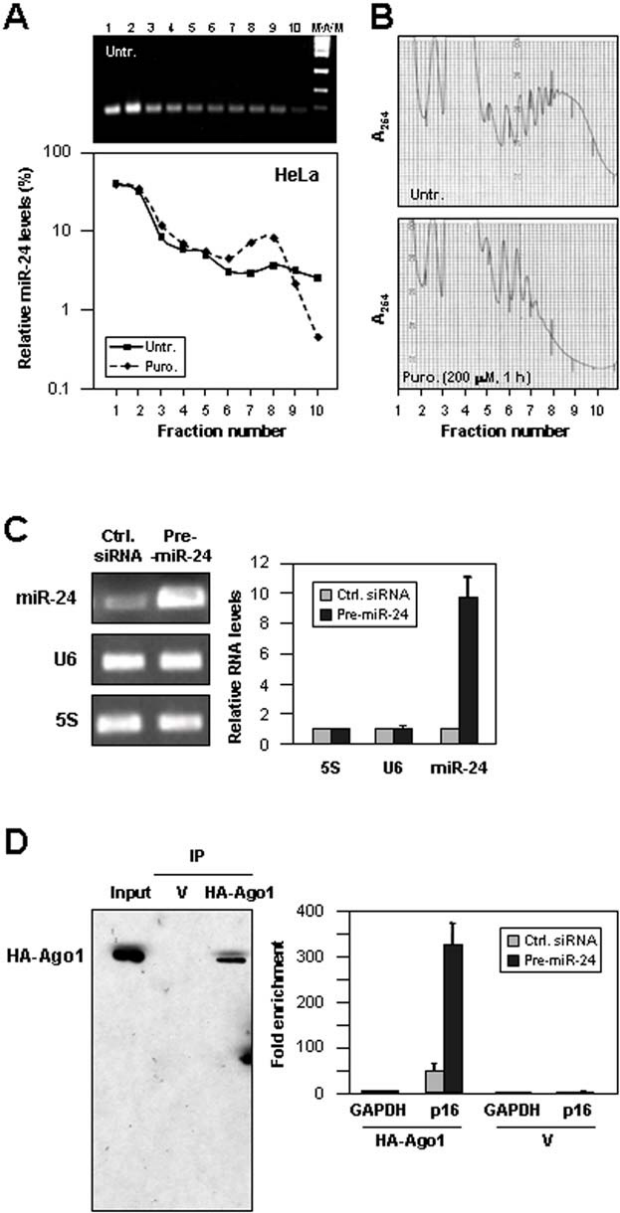
miR-24 associates with p16 mRNA and with actively translating polysomes in HeLa cells. (A) Cells were left untreated (*Untr.*) or treated with puromycin (*Puro.*, 200 µM, 1 hr); cytoplasmic lysates were prepared and fractionated through sucrose gradients. *Top*, representative miR-24 levels in each fraction, visualized after RT-PCR (30 cycles) and electrophoresis (1.5% agarose). *Bottom*, miR-24 levels in each fraction, quantified by RT-qPCR analysis and represented as % of the total miR-24 (semilogarithmic scale). (B) Representative gradient profiles. (C) By 48 hr after transfection of pre-miR-24 or control (Ctrl.) siRNA (100 nM), miR-24 and U6 snRNA levels were visualized on agarose gels (*left*), quantified by RT-qPCR, and normalized to 5S (*right*). Data (means+SD) represent the fold differences in miR-24 levels between the transfection groups. (D) HeLa cells were transfected with a plasmid expressing either pTRESNeo (V) or pIRESNeo-HA-Ago1 (HA-Ago1); the specific IP of HA-Ago1 was assessed by Western blot analysis of the IP reaction products. Forty-eight hr after transfection of HeLa cells with plasmid (V or HA-Ago1) and either control (siRNA) or pre-miR-24 RNA, RT-qPCR analysis was used to test the association of p16 mRNA with the RISC complex in the V and HA-Ago1 groups after HA IP. Graphs show the means+SD from 3 independent experiments.

We used HeLa cells to investigate how miR-24 regulated p16 expression. Using HeLa cells, polysome fractionation followed by RT-qPCR analysis revealed that, similarly to WI-38 HDFs, miR-24 was localized predominantly in fractions 1 and 2, and hence dissociated from the translational apparatus (Fig. 5A). However, a fraction of miR-24 was also present in association with translating polyribosomes, since puromycin treatment shifted the miR-24 distribution towards lighter gradient fractions (Fig, 5A,B). The significance of this distribution pattern and the precise location within sucrose gradients wherein miRNAs exert their translation inhibitory function remain to be elucidated. First, we tested the effect of overexpressing miR-24 in HeLa cells by transfecting pre-miR-24 and monitoring its abundance in cells by RT-qPCR (Fig. 5C). Evidence that miR-24 interacted with the p16 mRNA was then obtained using a method previously reported to study the functional effects of miRNAs on target mRNAs [29]. HeLa cells were co-transfected with a plasmid that expressed HA-Ago1 and with RNAs (Ctrl siRNA or pre-miR-24 RNA) for 24 hr, following which HA-Ago1 was immunoprecipitated (Materials and Methods). RT-qPCR analysis of the IP material revealed that the presence of p16 mRNA in the HA-Ago1 IP increased markedly after overexpressing miR-24 (Fig. 5D). Similarly, other miR-24 targets such as the CDK6 mRNA showed increased association with HA-Ago1 after overepressing miR-24, while CDK6 mRNA levels remained unchanged (Supplemental Fig. S5).

**Figure 6:**
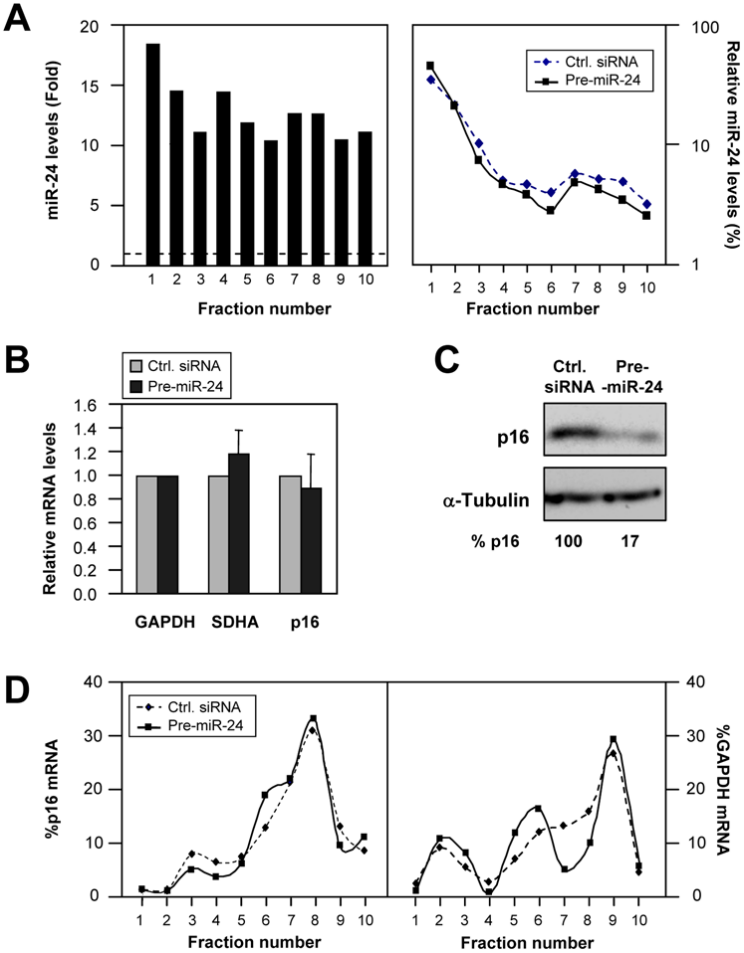
Increasing miR-24 reduces p16 levels in HeLa cells. (A) HeLa cells were transfected with either Ctrl. siRNA or pre-miR-24 (100 nM) and 48 hr later cytoplasmic lysates were prepared and fractionated through sucrose gradients. The levels of miR-24 were then measured by RT-qPCR in each of the fractions (Materials and Methods) and shown as relative (fold) miR-24 levels in the pre-miR-24 transfection group relative to the levels in the Ctrl. siRNA transfection group (*left*) and also as a percentage of the total miR-24 in each of the two transfection groups (*right*). (B) HeLa cells were transfected and processed as described in panel (A) except that the comparisons were made between populations transfected with AS-miR-24 and Ctrl. siRNA. The levels of mRNAs encoding p16 and housekeeping controls GAPDH (for normalization) and SDHA were calculated by RT-qPCR and represented (means+SD from 3 independent experiments) as fold differences relative to control transfected cells. (C) Representative Western blot analysis of p16 (and loading control α-Tubulin) levels in each transfection group. p16 protein signals were measured by densitometry and represented as a percent of the p16 levels in the control transfection group. (D) Representative distribution of p16 and GAPDH mRNAs on polysome gradients, calculated and shown as explained for Fig. 1C.

Overexpression of miR-24 in HeLa cells did not significantly alter the relative distribution of miR-24 on polysome gradients (Fig. 6A), nor did it influence the levels of p16 mRNA in the Ctrl. siRNA and pre-miR-24 transfection groups (Fig. 6B). However, p16 protein levels were markedly lower in the pre-miR-24 group relative to the control group (Fig. 6C). Since the p16 mRNA distribution profiles in polysome gradients from control and pre-miR-24 transfection groups were largely overlapping (Fig. 6D), the reduced p16 protein levels did not appear to result from lower p16 translational initiation. Instead, our collective results suggest that miR-24 overexpression reduces the elongation, rather than the initiation of p16 translation.

### Enhanced p16 Expression After Reducing miR-24 Function

**Figure 7:**
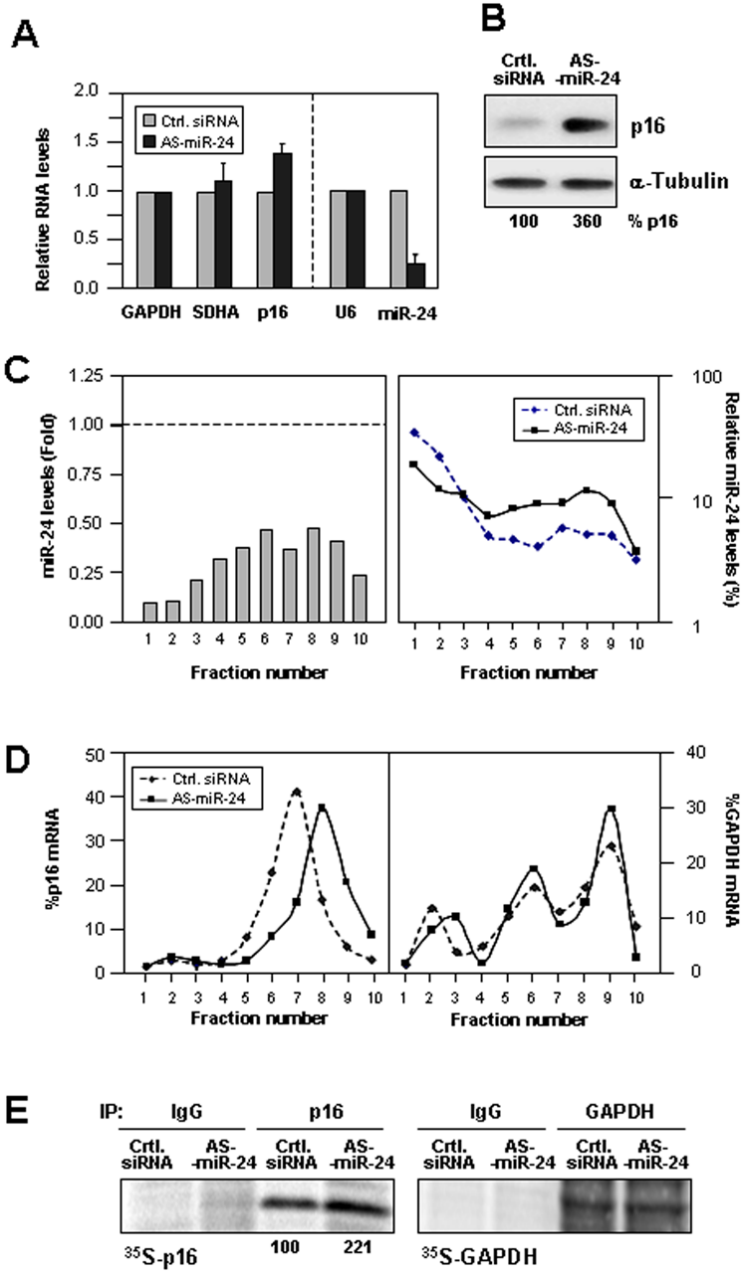
Lowering miR-24 increases p16 levels in HeLa cells. (A) Cells were transfected with either a control (Ctrl.) siRNA or antisense (AS) miR-24 (100 nM) and the levels of mRNAs encoding p16, and controls GAPDH (for normalization) and SDHA were calculated by RT-qPCR and represented as the means+SD from 3 independent experiments; the levels of miR-24 and U6 RNA were also measured. (B) Representative Western blot analysis of p16 (and loading control α-Tubulin) levels in each transfection group. p16 signals were measured by densitometry and represented as % p16 levels relative to the control transfection group. (C) The levels of miR-24 were measured by RT-qPCR in each of the fractions (Materials and Methods) and shown as relative miR-24 levels in the AS-miR-24 transfection group relative to the levels in the Ctrl. siRNA transfection group (*left*) and also as a percentage of the total miR-24 in each of the two transfection groups (*right*). Note that the abundance of miR-24 was preferentially reduced in fractions 1 and 2 (*left*), but the remaining miR-24 was found distributed among all of the fractions of the polysome gradient (*right*). Data are representative from three independent experiments yielding similar results. (D) The levels of p16 and control GAPDH mRNAs on polysome gradients were measured and represented as explained in the legend of Fig. 1C. (E) Nascent translation of p16 or (control) GAPDH was monitored following incubation of cells with L-[^35^S]methionine and L-[^35^S]cysteine for 15 min; after immunoprecipitation (IP) using either anti-p16 (*left*) or anti-GAPDH (*right*) antibodies along with control IgG, the incorporation of radiolabeled amino acids into newly synthesized p16 and GAPDH polypeptides was visualized after SDS-PAGE and quantified using a PhosphorImager; the % change in p16 nascent translation is shown.

Further analysis of the influence of miR-24 upon the translation of p16 was conducted by introducing an transcript antisense (AS) to miR-24. By 48 h after transfection of 100 nM AS-miR-24, the levels of miR-24 were markedly reduced, suggesting that the RNA duplex promoted the degradation of miR-24 (Fig. 7A). AS-miR-24-overexpressing HeLa cells displayed modest increases in p16 mRNA levels compared with control (Ctrl.) siRNA-transfected populations (Fig. 7A), although the p16 mRNA half-life (>12 h) appeared unaffected by the reduced miR-24 levels (not shown). By contrast, p16 expression increased >threefold (Fig. 7B). Despite its reduced abundance, the remaining miR-24 showed a comparable polysome gradient distribution between the two transfection groups (Fig. 7C). The translational status of p16 mRNA was tested by comparing its relative distribution in sucrose gradients prepared from both transfection groups. As shown, AS-miR-24-expressing cells exhibited a moderate but distinct shift towards the actively translating fractions, indicating that p16 mRNA associated with larger polysomes in these cells (Fig. 7D), and suggesting that translational initiation was enhanced after miR-24 levels were reduced. Together with plausible changes in the rates of translational elongation, as discussed above (Fig. 4E), AS-miR-24 caused a marked elevation in p16 expression levels (Fig. 7B).

Importantly, the notion that p16 translation was comparatively higher was supported by measuring *de novo* p16 biosynthesis after a brief (15-min long) incubation period with ^35^S-methionine and ^35^S-cysteine, immediately followed by immunoprecipitation (IP) reactions using anti-p16 or control IgG antibodies. The radiolabeled signals revealed >twofold higher nascent p16 translation in the AS-miR-24 transfection group (Fig. 7E), while the nascent translation of a control housekeeping protein (GAPDH) was unaffected. These findings indicated that miR-24 contributed to lowering the translation rate of p16, and suggested that a reduction in translational initiation also contributed to this inhibitory effect. It is formally possible that the changes in miR-24 levels and function (by pre- or AS-miR-24 molecules) do not affect p16 translation and instead influence the stability of p16 protein. This is a less plausible mode of action for miR-24, given the paucity of evidence that p16 levels are controlled through regulated proteolysis. In a single study, p16 degradation was found to be governed by the ubiquitin-proteasome system in a density-dependent manner [30]; in this regard, cell density was virtually the same among the HeLa and WI-38 transfection groups. Using cycloheximide, we did not detect any influence of miR-24 on p16 protein half-life (not shown).

### Heterologous Reporter Analysis of miR-24 Influence on p16 Expression

**Figure 8:**
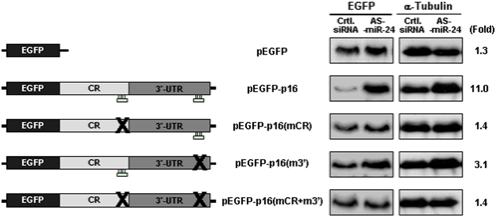
Analysis of p16 predicted sites of miR-24 association using a heterologous reporter. Schematic (*left*) and names (*center*) of the EGFP and chimeric EGFP-p16 reporter plasmids tested (Materials and Methods). By 48 hr after co-transfection of HeLa Tet-off cells with Ctrl. siRNA or AS-miR-24, along with the plasmids indicated, the levels of EGFP expressed from each reporter vector was assessed; shown are representative Western blotting signals and quantification (*Fold*, AS-miR-24 vs. Ctrl. siRNA) of EGFP reporter levels in each transfection group after normalization to α-tubulin signals.

The influence of AS-miR-24 on p16 translation was further tested by employing a heterologous reporter system that studies the expression of d2EGFP (Clontech, Materials and Methods), a short-lived variant of EGFP which is uniquely suited for this analysis. The effect of sequences within the p16 mRNA on d2EGFP protein expression was studied by using a previously reported tTA-regulated construct [17] which expresses a chimeric mRNA comprising the EGFP coding region and the entire p16 CR and 3′UTR (EGFP-p16 mRNA). HeLa Tet-off cells were transfected transiently with a vector to express EGFP mRNA or with one of four vectors to express EGFP-p16 mRNA bearing intact miR-24 target CR and 3′UTR sequences, mutated CR (mCR), mutated 3′UTR (m3′) or both mutations (mCR+m3′), as shown in Fig. 8 (schematic). By 48 hr after transfection, the expression of both EGFP and EGFP-p16 mRNAs remained unchanged as a function of AS-miR-24 (Supplemental Fig. S7). By contrast, the levels of EGFP protein expressed from each transcript was markedly different: EGFP protein expressed from the control EGFP reporter was unchanged between the control and AS-miR-24 transfection groups, while EGFP expressed from the EGFP-p16 chimeric mRNA was significantly higher (∼11-fold) in the AS-miR-24 group than in the control group. Mutation of the p16 CR site predicted to be targeted by miR-24 (mCR) or both the CR and 3′UTR sites (mCR+m3′) effectively abrogated this induction in EGFP expression, while mutation of only the 3′UTR (m3′) had a partial effect (∼3-fold induction), as shown in Fig. 8. These results strongly support the view that miR-24 influences p16 expression through the predicted CR and 3′UTR target sites.

### miR-24 Appears to Suppress the Initiation and Elongation of p16 Translation

Collectively, our results indicate that miR-24 suppresses p16 translation in a cancer cell model (HeLa) and in a model of replicative senescence (WI-38 HDFs). The finding that miR-24 suppresses p16 translation but does not appear to influence p16 mRNA levels agrees with growing evidence that mammalian miRNAs more commonly suppress protein biosynthesis than they promote target mRNA degradation. The evidence presented here provides mechanistic support for the notion that miR-24 suppresses p16 production by inhibiting both the *initiation* and *elongation* of p16 translation.

Supporting the possibility that p16 translation *initiation* was suppressed by miR-24 are data showing that a reduction in miR-24 function by AS-miR-24 caused a modest shift in p16 mRNA towards heavier polysomes (Fig. 7D). This rightward shift in polysome size indicates that, on average, individual p16 mRNA molecules were associated with a higher number of ribosomes and were thus expected to be translated more actively. The miRNA-mediated silencing of gene expression has been linked to the recruitment of target mRNAs to P-bodies; these foci are implicated in mRNA degradation [31], and also in translational repression as they lack translational constituents and contain protein components of RISC (such as Ago1) that interact directly with miRNAs [32]. The suppression of translation by miRNA/RISC has been linked to the presence of the eIF6 anti-association factor (which prevents the assembly of the 80S ribosome) [33]. It remains to be established whether miRNA/RISC also suppresses translational initiation by causing the mRNA to exit translation, by recruiting the mRNA to P-bodies, or by promoting the association of RBPs that transport the mRNA to P-bodies [19].

The notion that miR-24 might also suppress the *elongation* phase of p16 translation is supported by our results that ectopic miR-24 overexpression markedly reduced p16 protein levels without changing p16 mRNA levels or distribution on polysome gradients (Fig. 6D). This mechanism of miRNA action is reminiscent of that described for the *C. elegans* miRNA lin-4, which silenced lin-14 and lin-28 mRNAs without changing their polysomal distribution [34], [35]. Overexpression of miR-24 elevated its abundance in all of the heavy polysome fractions (Fig. 6A), a finding that agreed with miR-24's suppression of the elongation phase of mature, heavy polysomes. By contrast, downregulation of miR-24 (Fig. 7A) preferentially reduced miR-24 abundance in non-polysomal gradient fractions (Fig. 7C); this distribution pattern could preferentially facilitate the loading of ribosomes (initiation step) and have a lesser influence on the elongation of formed polysomes, although this hypothesis awaits experimental analysis. The miRNA-mediated suppression of elongation and termination of translation are less well characterized, although recent studies indicate that miRNAs can block elongation of translating polysomes by causing them to cease translation and ‘drop-off’ [24]. Moreover, miRNA/RISC complexes could influence all steps in translation (initiation, elongation, termination) in ways that the distribution on polysome profiles could remained unchanged, although it must be noted that if only elongation or termination were inhibited, the mRNAs would be expected to form *larger* polysomes.

### Concluding remarks

Evidence is mounting that an integrated set of mechanisms controls p16 levels. Transcriptionally, p16 expression is dictated by changes in chromatin and transcription factors, and posttranscriptionally, it is shaped through changes in mRNA stability and translation. Future study of the physical and functional connections among posttranscriptional *trans* acting factors (miRNAs and RBPs) operating upon the p16 mRNA and other transcripts promises to be a particularly exciting endeavor. For instance, the co-transcriptional loading of *trans* factors can link transcriptional events with subsequent splicing, transport, stability, and translation [36], *trans* factors are implicated in mRNA stability and translation decisions made at P-bodies and other cytoplasmic structures [37], [38], and are anticipated to interact richly upon the target mRNA [39]. For genes playing pivotal cellular functions, such as p16 and other tumor suppressors and senescence-associated proteins, such multi-leveled, complex regulatory networks are expected. The resulting system of checks and balances will ensure the precise abundance, location, and timing of protein production.

## Materials and Methods

### Cell Culture and Transfections

Human cervical carcinoma HeLa cells were cultured as described [40]. Early-passage (∼20–25 pdl) and late-passage (∼50–55 pdl) human WI-38 human diploid fibroblasts (HDFs, Coriell Cell Repositories) were cultured as described [17]. Synthetic 2′O-methyl antisense (AS-miR-24) or pre-miR-24 oligonucleotides (Ambion) were transfected at a final concentration of 100 nM; oligonucleotides and plasmids were transfected with Lipofectamine 2000 (Invitrogen). Plasmids pTRESNeo (V, from Clontech) or pIRESNeo-HA-Ago1 (HA-Ago1, from Addgene) were transfected (2 µg per plate) to study the association of miR-24 with mRNAs in the RISC complex.

HeLa Tet-off cells (Clontech) were transfected with the parent reporter vector pTRE-d2EGFP (*pEGFP*), or with pTRE-d2EGFP-p16(CR+3′UTR) (*pEGFP-p16*), which comprises the p16 CR and 3′UTR wild-type [17] or mutated sequences of the predicted CR and/or 3′UTR miR-24 sites. The reporter vectors used in Fig. 8 are as follows: *pEGFP* – parent reporter vector pTRE-d2EGFP; *pEGFP-p16* – plasmid pTRE-d2EGFP-p16(CR+3′UTR), which comprises the wt p16 CR and 3′UTR [17]; *pEGFP-p16(mCR)* – plasmid pTRE-d2EGFP-p16(CR+3′UTR), in which the predicted p16 CR miR-24 site was mutated (m) from TCCTGGCTGAGGAGCTGGGCCA to TCCTGGCTGAGGAGCTGCGA CA by site-directed mutagenesis; *pEGFP-p16(m3*′*)* – plasmid pTRE-d2EGFP-p16(CR+3′UTR), in which the predicted p16 3′UTR miR-24 site was mutated (m) from GTTACTGGCTTCTCTTGAGTCA to GTTACTGGCTTCTCTTGCGGCA by site-directed mutagenesis; *pEGFP-p16(mCR+m3*′*)* – plasmid pTRE-d2EGFP-p16(CR+3′UTR), in which both of the predicted miR-24 target sites on the p16 CR and 3′UTR were mutated as indicated above. Briefly, HeLa tet-off cells were contransfected with 0.5 µg of plasmid pTRE-d2EGFP (with or without the binding site for miR-24) along with either Ctrl. siRNA or AS-miR-24, using Lipofectamine 2000; 48 hr later, cells were harvested in RIPA buffer and samples subjected to SDS-PAGE and Western blot analysis to detect EGFP and α-Tubulin. Transcription was not shut off at any point during the analysis.

### Prediction of p16 mRNA as a target of miR-24

The p16 mRNA was predicted to be the target of miR-24 at two sites (CR and 3′UTR) after performing computational analyses using two different programs, Miranda and RNA22. Using RNA22 (http://cbcsrv.watson.ibm.com/rna22.html) and the sequences of p16INK4A and miR-24, the program returned the two hits shown in Fig. 3A (in the CR and the 3′UTR) and in Supplemental Figure S7. The default stringency settings were used: maximum number of allowed UN-base paired bases = 0 in seed/nucleus of 7 nucleotides, and minimum number of paired-up bases in heteroduplex = 14; maximum folding energy for heteroduplex (Kcal/mol = −25).

### Polyribosome Fractionation

Cells were incubated with cycloheximide (100 µg/ml, 15 min) and cytoplasmic lysates (500 µl) were fractionated by centrifugation through 10–50% linear sucrose gradients and divided into 10 fractions for analysis, as described [40].

### RNA Isolation and analysis

Total RNA was isolated with the Trizol reagent (Invitrogen). Conventional and quantitative (or real-time, *q*) RT-PCR were done as described [40]. Briefly, 1 µg of total RNA or 50% of RNA isolated from each gradient fraction were reverse transcribed using random hexamers and SSII RT (Invitrogen); the resulting cDNA was amplified by PCR using gene-specific primer pairs; mature miR-24, 5S rRNA, and U6 snRNA were quantified using mirVana miRNA primer sets and qRT-PCR miRNA detection kit (Ambion).

MicroRNA microarray analysis was performed by using the miRCURY™ LNA Array (version 8.1) microRNA Profile Service from Exiqon (Vedbaek, Denmark). Total RNA (10 µg) prepared from Y (pdl 25) and S (pdl 54) was used. Three independently prepared sample sets were used for microarray analysis; miRNAs were considered to be differentially expressed when they were upregulated or downregulated in at least two experiments.

### Western Blotting

Proteins were resolved by 14% SDS-PAGE and transferred to PVDF membranes (Invitrogen). Primary antibodies recognized p16, α-tubulin, EGFP (Santa Cruz Biotech.) or β-actin (Abcam); after secondary antibody incubations, protein bands were detected by ECL-plus (Amersham).

### Analysis of Nascent Protein

*De novo* p16 or GAPDH protein synthesis was measured by incubating HeLa cells with 1 mCi L-[^35^S]methionine and L-[^35^S]cysteine (Easy Tag EXPRESS, NEN/PerkinElmer) for 15 min, followed by lysis with RIPA buffer and IP with 10 µg anti-p16 or anti-GAPDH antibodies (Santa Cruz Biotech.); IgG was used in control IP reactions. Beads were washed in RIPA buffer and the IP material was resolved by 14% SDS-PAGE, transferred onto PVDF membranes, and visualized and quantified using a PhosphorImager.

## Supporting Information

Figure S1 (0.02 MB PDF)

Figure S2 (0.01 MB PDF)

Figure S3 (0.15 MB PDF)

Figure S4 (0.46 MB PDF)

Figure S5 (0.03 MB PDF)

Figure S6 (0.04 MB PDF)

Figure S7 (0.01 MB PDF)
