# Supplementary material for: p16INK4a Translation Suppressed by miR-24
Source: PLoS One. 2008 Mar 26;3(3):e1864. doi: 10.1371/journal.pone.0001864 (PMC2274865; doi:10.1371/journal.pone.0001864)
Supplement: Figure S1 — (0.02 MB PDF) [file pone.0001864.s001.pdf]

```
From offset 559 to 580 | Folding energy = -37.900002 Kcal/mol
5'<-- target -->3'     -linker-    5'<-- microRNA -->3'
ACCTGGCTGAGGAGCTGGGCCA GCGGGACGC UGGCUCAGUUCAGCAGGAACAG
.((((.((((...(((((((((---------)))))))))))))))))).....
A----CCTGGCTGAGGAGCTGGGCCA
     |||| ||||   |||||||||
GACAAGGAC-GACU---UGACUCGGU
```

```
From offset 1074 to 1095 | Folding energy = -26.799999 Kcal/mol
5'<-- target -->3'     -linker-    5'<-- microRNA -->3'
GTTACTGGCTTCTCTTGAGTCA GCGGGACGC UGGCUCAGUUCAGCAGGAACAG
(((.((.(((....((((((((---------))))))))...))).))))).. 
--GTTACTGGCTTCTCTTGAGTCA
  ||| || |||    ||||||||
GACAA-GGACGACUU-GACUCGGU
```

Supplemental Figure S1. RNA22 analysis (http://cbcsrv.watson.ibm.com/rna22.html)**.** RNA22 analysis of the p16INK4A and miR-24 sequences yielded the predicted sites of miR-24 association with the p16 mRNA [CR (559-580) and 3'UTR (1074-1095)]. The default stringency settings were used:

- maximum number of allowed UN-base paired bases = 0 in seed/nucleus of 7 nucleotides
- minimum number of paired-up bases in heteroduplex = 14
- maximum folding energy for heteroduplex (Kcal/mol = -25)
