# Supplementary material for: p16INK4a Translation Suppressed by miR-24
Source: PLoS One. 2008 Mar 26;3(3):e1864. doi: 10.1371/journal.pone.0001864 (PMC2274865; doi:10.1371/journal.pone.0001864)
Supplement: Figure S2 — (0.01 MB PDF) [file pone.0001864.s002.pdf]

miRNA	Fold
hsa-miR-146b	**8.8**
hsa-miR-492	**3.9**
hsa-miR-584	**3.7**
hsa-miR-663	**2.4**
hsa-miR-638	**2.2**
hsa-miR-602	**2.2**
hsa-miR-210	**2.1**
hsa-miR-572	**2.1**
hsa-miR-498	**2.0**

Supplemental Figure S2. miRNAs showing increased expression levels in senescent cells. Three independent preparations of total RNA from early-passage (Young, pdl 25) and from late-passage (Senescent, pdl 54) WI-38 cells were subjected to microarray analysis (Exiqon, Materials and Methods). Nine miRNAs showing increased levels are listed.
