# Supplementary material for: p16INK4a Translation Suppressed by miR-24
Source: PLoS One. 2008 Mar 26;3(3):e1864. doi: 10.1371/journal.pone.0001864 (PMC2274865; doi:10.1371/journal.pone.0001864)
Supplement: Figure S4 — (0.46 MB PDF) [file pone.0001864.s004.pdf]

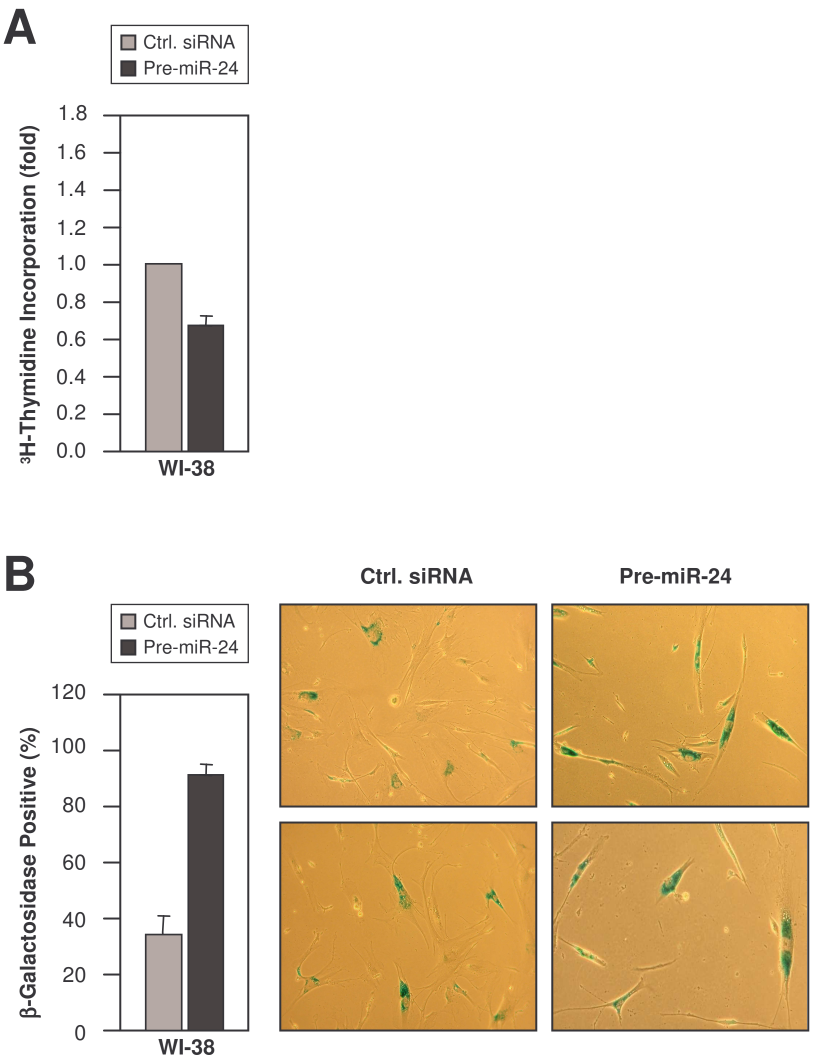

Supplemental Figure S4. Analysis of ^{3}H-Thymidine incorporation and senescence-associated β-galactosidase activity after increasing miR-24 leves in WI-38 cells. (A) Pre-senescent WI-38 (Pdl 48) cells were transfected with Pre-miR-24 (100 nM) and 48 hr later they were incubated with 2 μCi ^{3}H-Thymidine for 16 hr, whereupon ^{3}H-Thymidine incorporation was measured in both transfection groups using standard procedures. Data were calculated as ^{3}H-Thymidine incorporation in Pre-miR-24-transfected cells relative to that in Ctrl. siRNA and shown as fold change. Data are the means + SD from 3 independent experiments. **(B)** WI-38 cells (Pdl 48) were transfected with either Ctrl. siRNA or Pre-miR-24 (100 nM) every 4 days; two weeks later, the levels of a senescence-associated β-galactosidase activity was assessed by using a kit from Cell Signaling. The number of β-galactosidase-positive cells was quantified from 3 different experiments (means +SEM are plotted); two representative fields from each transfection group are shown.
