# Supplementary material for: p16INK4a Translation Suppressed by miR-24
Source: PLoS One. 2008 Mar 26;3(3):e1864. doi: 10.1371/journal.pone.0001864 (PMC2274865; doi:10.1371/journal.pone.0001864)
Supplement: Figure S5 — (0.03 MB PDF) [file pone.0001864.s005.pdf]

A

	Miranda	RNA22
CDK6	2918-2941	1704-1725
CDK6	3643-3664	2920-2941
CDK6	10877-10894	2639-2660
CDK6		3571-3592
CDK6		6371-6392

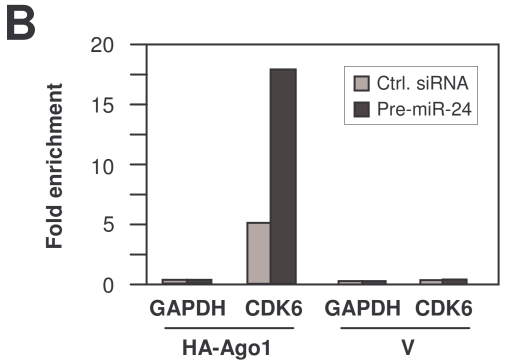

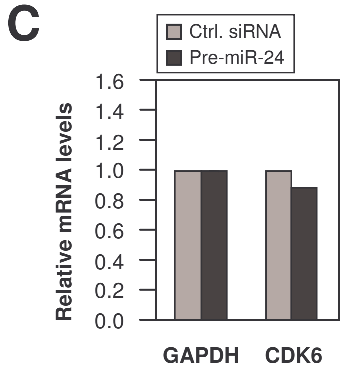

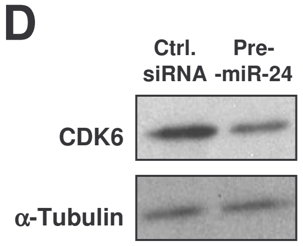

Supplemental Figure S5. CDK6 is a functional target of miR-24. (A) CDK6 mRNA showing predicted miR-24 binding sites (5 using RNA22, 3 using Miranda). **(B)** Forty-eight hr after transfection of HeLa cells with plasmid (V or HA-Ago1) and either control (siRNA) or pre-miR-24 RNA, RT-qPCR analysis was used to test the association of CDK6 mRNA with the RISC complex in the V and HA-Ago1 groups in the IP reactions. **(C)** CDK6 and GAPDH mRNA levels in the transfection groups were unchanged. **(D)** Western blot analysis of CDK6 expression after increasing miR-24 levels.
