# Supplementary material for: p16INK4a Translation Suppressed by miR-24
Source: PLoS One. 2008 Mar 26;3(3):e1864. doi: 10.1371/journal.pone.0001864 (PMC2274865; doi:10.1371/journal.pone.0001864)
Supplement: Figure S6 — (0.04 MB PDF) [file pone.0001864.s006.pdf]

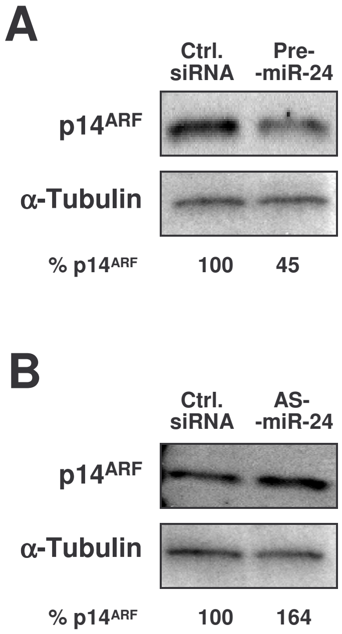

Supplemental Figure S6. Levels of p14^{ARF} after modulating miR-24 levels. (A) Effect of overexpressing miR-24 on p14^{ARF} expression in HeLa cells. Cells were transfected with either Ctrl. siRNA or Pre-miR-24 or (100 nM each) as described in Fig. 2. Forty-eight hr later, lysates were prepared in order to assess the levels of p14^{ARF} and loading control α-Tubulin by Western blot analysis and quantify the signals by densitometry. Data are representative of two independent experiments. **(B)** Effect of reducing miR-24 levels on p14^{ARF} expression in HeLa cells. Cells were transfected with either Ctrl. siRNA or Pre-miR-24 or (100 nM each) as described in Fig. 3. Forty-eight hr later, lysates were prepared and the levels of p14^{ARF} and loading control α-Tubulin were assessed by Western blot analysis and quantified. Data are representative of two independent experiments. The anti-p14^{ARF} antibody was from Santa Cruz Biotech.
