# Supplementary material for: p16INK4a Translation Suppressed by miR-24
Source: PLoS One. 2008 Mar 26;3(3):e1864. doi: 10.1371/journal.pone.0001864 (PMC2274865; doi:10.1371/journal.pone.0001864)
Supplement: Figure S7 — (0.01 MB PDF) [file pone.0001864.s007.pdf]

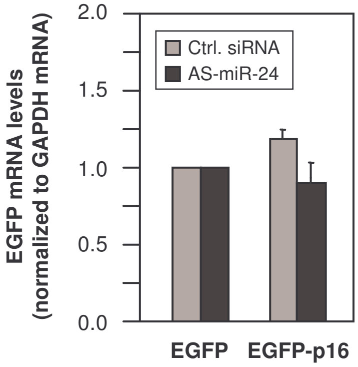

Supplemental Figure S7. Reporter EGFP mRNA levels. By 16 hr after transfection of HeLa Tet-off cells with either Ctrl. siRNA or AS-miR-24, along with plasmids pEGFP-p16 or pEGFP (described in Fig. 3E and in the Experimental Procedures section), EGFP and EGFP-p16 mRNA levels were measured by RT-qPCR analysis. Data are the means +SD from 3 independent experiments.
